# Bone–Ti-Alloy Interaction in Hip Arthroplasty of Patients with Diabetes, Dyslipidaemia, and Kidney Dysfunction: Three Case Reports and Brief Review

**DOI:** 10.3390/medicina61122228

**Published:** 2025-12-17

**Authors:** Cosmin Constantin Baciu, Ana Maria Iordache, Teodoru Soare, Nicolae Catalin Zoita, Cristiana Eugenia Ana Grigorescu, Mircea Bogdan Maciuceanu Zarnescu

**Affiliations:** 1 University of Medicine and Pharmacy Carol Davila (UMFCD), Dionisie Lupu Street, No. 37, Sector 2, 050474 Bucharest, Romania or cosminbaciu82@gmail.com (C.C.B.); or bmaciuceanu@gmail.com (M.B.M.Z.); 2 Clinical Emergency Hospital (SCUB), Floreasca Route, No. 8, Sector 1, 014461 Bucharest, Romania; 3 National Institute of Research and Development for Optoelectronics—INOE 2000, 077125 Magurele, Romania; 4 Department of Pathology, Faculty of Veterinary Medicine, University of Agronomic Sciences and Veterinary Medicine of Bucharest, Splaiul Independentei Street, No. 105, Sector 5, 050097 Bucharest, Romania; 5 SC Histovet SRL, Histovet-Veterinary Laboratory, Sirenelor Street, No. 81, Sector 5, 050855 Bucharest, Romania

**Keywords:** hip arthroplasty, titanium alloy prostheses, dyslipidaemia, diabetes, kidney dysfunction, bone-Ti alloy interaction, bone health, post-traumatic panniculitis, scanning electron microscopy, electrochemistry

## Abstract

*Background and Objectives*: Organ dysfunctions affect the quality of bone and body fluids. This case report seeks links between the underlying conditions of three patients undergoing hip arthroplasty (HA) with uncemented implants, the quality of their bones, and their Ti-6Al-4V orthopaedic implants, on different time spans. Femoral stems are investigated. A brief review supports our findings. *Materials and Methods*: Cases: two women (F1 35+, F2 80+), and one man (M 65+), all having diabetes, dyslipidaemia, and kidney dysfunction. Samples: a segment of a broken 7-year-old stem, bone with a metallic layer, soft tissue, segments of one spare stem, and synthetic plasma enriched with glucose and urea according to the biochemistry tests of the respective patients. Vast studies show that cholesterol influences bone quality only. The stem pieces were ultrasonicated for 7 h at 37 °C in synthetic plasma. Scanning electron microscopy (SEM), energy dispersive X-ray spectroscopy (EDX), and profilometry investigated the Ti-alloy samples, electrochemistry analysed the post-sonication plasma, and histopathology examination was performed on the soft tissue remnants on the broken stem. *Results*: EDX show that all stem samples are Ti-6Al-4V with minute additions of other elements and hydroxyapatite (HAp) coating. SEM and profilometry analysis are consistent for the roughness in the outer layers of the stems. Electrochemistry on the bone fragment shows migration of vanadium during the 6 months since fracture to revision for M. *Conclusions*: Stems in altered synthetic plasma are affected by glucose and urea. Metal migration from the prostheses can occur through the chemical interactions between body fluids with abnormal biochemistry and the orthopaedic prostheses, favoured by cracks and concurring with wear following friction during usual movements. Cholesterol influences on the bone quality.

## 1. Introduction

Hip arthroplasty (HA) is a surgical reconstruction of the hip joint using artificial material, including total hip arthroplasty (THA), when the femur’s head and the acetabulum are replaced. Orthopaedic surgery involves various kinds of implants, whose interfaces with the bone tissues, and their surroundings, are dramatically influenced by the characteristics of the implant, e.g., geometry, material, surface morphology, topology, mechanical, physical, chemical properties [1,2,3,4,5,6,7,8,9], and by interaction with the living body sometimes presenting dysfunctions of important internal organs, such as pancreas, kidneys, parathyroid, liver, and heart [10,11,12,13,14,15]. Wear, corrosion, and bacteriogenesis are recognised among the chief mechanisms leading to revision at some instant. New material combinations have been developed to diminish friction and wear, along with improved simulations to reproduce clinical conditions, issued from patients’ activities, and mimic their related underlying conditions [12,13,14,15]. Biocompatibility is the primary request from implants, and it includes the characteristics mentioned above. In addition, the interaction with plasma proteins, and cellular blood components, must occur at the equilibrium, in such a way that the biological environment, e.g., bone, bone marrow, body fluids, would not alter the material properties.

Titanium, and its alloys, such as Ti-6Al-4V, have become the most preferred materials in orthopaedics prosthetics [2,9,15,16,17,18]. The tuning of implant surfaces through the adjustment of porosity, and the growth of oxide coatings, especially hydroxyapatite (HAp), has led to important improvements in terms of preventing the migration of metal ions, and to enhancing osseointegration [5,6,9]. Even though titanium is known for its low toxicity, high biocompatibility, and exceptional mechanical properties (Young modulus, wear resistance, friction coefficient at bone/alloy interface), implants can be subject to corrosion when in contact with fluids in the human body. This can occur, for instance, when inflammatory states are present, or when HCl compounds are signalled [17,19]. In this work, we bring to the readers’ attention three cases of patients suffering simultaneously from diabetes, dyslipidaemia, and kidney dysfunction, undergoing HA with uncemented Ti-alloy, with an aim towards understanding better some interconnections between the underlying conditions of patients, the quality of their bones, and their orthopaedic implants, on different time spans. We refer to femoral stems only.

## 2. Materials and Methods

### 2.1. Three Case Reports

The cases reported here were two women (F1 and F2) and one man (M), who were admitted to the Clinical Emergency Hospital Bucharest (SCUB) for HA. Their description is summarised in Table 1.

**Figure 1:**
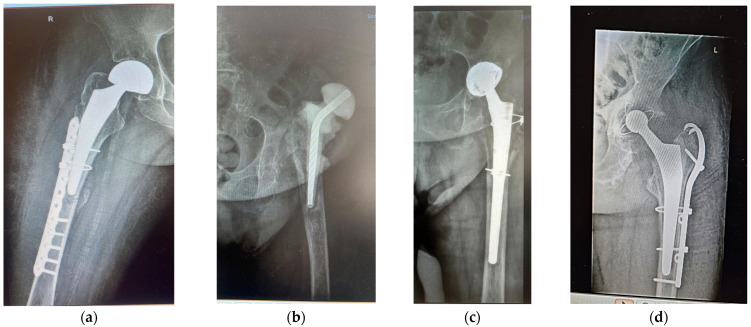
Anterolateral radiographs of (**a**) patient F1 (primary HA; hemiarthroplasty with bipolar prosthesis); (**b**,**c**) patient F2, AHT revision after 15 years, with antibiotic spacer; (**d**) patient M, revision after 7 years, because of periprosthetic fracture and stem cracked 6 months before revision.

Case 1. A woman (F1) over 35 years old was admitted to the Clinical Emergency Hospital Bucharest (SCUB) with a basicervical femoral neck fracture (BCFNF), right side, following an unspecified fall on a flat surface. Her underlying conditions were as follows: Type 1 diabetes (insulin dependency); renal failure end stage (dialysis dependency); and essential hypertension. BCFNFs are rare, about 1.8% of all proximal femoral fractures, according to results from the comprehensive critical review by Jun-Il Yoo et al. [20]. Though F1 is a young patient, and BCFNFs are specific to the elderly, caused by low energy impact, her underlying conditions have contributed to bone weakness. In their review, which considers 15 studies, Jun-Il Yoo et al. conclude that definitions of BCFNF vary greatly, and, in consequence, treatment methods and their results also vary greatly. The treatment option for F1 was hemiarthroplasty with a bipolar prosthesis. During surgery, the patient suffered a periprosthetic fracture (Figure 1a) that needed cable fixation.

Case 2. A woman (F2) aged over 80 suffered an unspecified fall while performing housework at her home, resulting in a periprosthetic fracture of her left hip. The patient was admitted to the Clinical Emergency Hospital Bucharest (SCUB) for revision. F2 had suffered a left side THA 15 years earlier, at a different clinic, with a Zimmer implant. High blood pressure (AHT), dyslipidaemia, diabetes, hyper-uraemia, and hypothyroidism were her comorbidities. During the revision surgery, F2 was treated for prosthetic joint infection with an antibiotic spacer for three months. Prosthetic joint infections are mainly treated through a two-stage process [21,22], where infected implants are removed and antibiotic spacers are placed to preserve the length. When the site is free of infection, revision is performed. Figure 1b,c show the anteroposterior radiographs of F2’s revision THA.

Case 3. A over 65-year-old man (M) was admitted to the Clinical Emergency Hospital Bucharest (SCUB) for revision of a 7-year-old implant (THA). The patient had fallen on a hard surface and suffered a periprosthetic fracture. The stem cracked. M presented himself to hospital 6 months after the fracture was produced. His underlying conditions were very complex: AHT third degree, congestive heart failure, old myocardial infarction, coronary artery disease (CAD), atrioventricular block (AV) first degree, right bundle branch block (RBBB), dyslipidaemia (mixed hyperlipidaemia), and azotaemia. The cracked stem was extracted, and THA revision was performed using Zimmer Biomet Ti6Al4V stem (Figure 1d). Remnants of bone and soft periprosthetic tissue from the retrieved stem were collected for further investigations. The bone fragment exhibited a thin metal layer.

Anterolateral radiographs of the three cases are shown in Figure 1. F1 had a right-sided fracture, with primary HA (hemiarthroplasty with bipolar prosthesis). During surgery, a new fracture occurred, imposing supplementary fixation with cables. F2 and M underwent revisions on their left sides. All prosthetic elements were from Zimmer Biomet Romania SRL, Splaiul Independentei 319L, Paris Office Building, Corp A1, Ground Floor, Bucharest, Romania.

### 2.2. Disorders in General Functions of the Body and Bone Health

As observed from the case presentation, the three patients share several underlying conditions:

F1, F2, M: AHT, diabetes, renal dysfunction;

F2, M: AHT, diabetes, dyslipidaemia, renal disfunction;

All of them primarily influencing the bone health.

A recent review by Panagiotis Anagnostis et al. [13] underlines the role of dyslipidaemias in atherosclerotic cardiovascular diseases, as well as in both bone, and vascular, mineralisation, showing the strong link between dyslipidaemias and osteoporosis. The study covers the worldwide population [23,24,25], focusing however on elderly, postmenopausal women, and women in general. Nevertheless, disturbances in the metabolism of lipoproteins and oxidative stress, and increased parathyroid hormone, enhance the processes of bone demineralisation, thus favouring fractures.

Diabetes mellitus is a major metabolic disorder, resulting from abnormal insulin production and action, manifested through hyperglycaemia. Type 1 diabetes mellitus is an auto-immune disease, where the islet cells in the pancreas are attacked by the immune system of the body and insulin is not produced. In adults, this disease results in the drastic modification of bone structure, mainly in the femur [26]. Type 2 diabetes mellitus is caused by the insufficient use of insulin, leading to insulin resistance. One consequence is the drastic decay of bone mineral density. Diabetes, both type 1 and type 2, influences bone health dramatically, lowering its mineral density, leading to the degeneration of its structure, and hence to frailty. There is a great number of studies, meta-analyses, and reviews dealing with the characteristics and pathogenesis, of diabetes mellitus, their consequences on bone health, and various ways of treatment. A very well organised work is the narrative review by Bo Wu et al. [27]. Diabetes, AHT, and kidney dysfunction are linked. High levels of glucose in blood destroy the filtering units in kidneys, thus progressively decreasing their filtration function, leading eventually to end stage renal failure (see patient F1). AHT and cardiovascular disease (including dyslipidaemias) diminish blood flow to the kidneys; the blood vessels become narrow and the pressure increases [28]. Patients with chronic kidney disease are at higher risk of fractures, especially hip fractures, as compared to healthy ones, because of changes in bone turnover, mineralization, and volume [29,30,31,32].

The three cases are anticipated to demonstrate specific interactions between compromised bone quality and titanium alloy prostheses.

### 2.3. Bone–Titanium Alloy Interaction—Study on Retrieved Prostheses, Ex Vivo Direct Bone Samples, and Simulated Body Fluids

**Figure 2:**
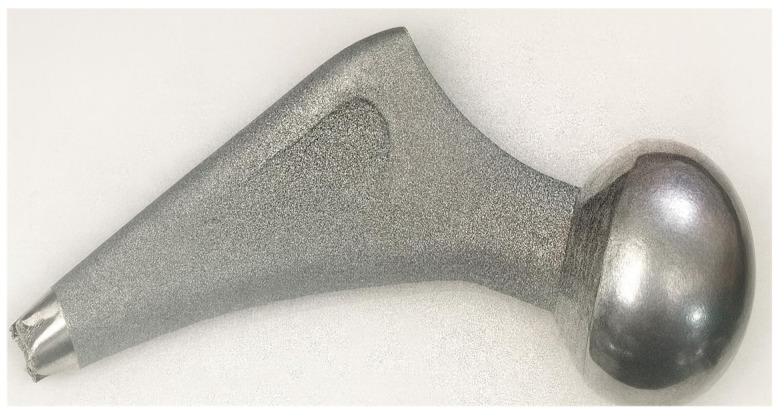
Stem 1 was retrieved from the femur of patient M.

Three stems made of Ti-alloys, and coated with HAp (Zimmer Biomet, Medical International, Romania), shown in Figure 2, have been investigated. The broken stem had lasted for 7 years in the femur of patient M, until he fell on a hard surface 6 months before presentation to hospital with a diaphyseal femoral fracture, for THA revision. Fragments of soft tissue, and a small piece of bone with a metallic thin layer, were collected from the broken stem and investigated.

In Table 2, the samples investigated in this work are concisely described.

#### 2.3.1. Stems in Synthetic Plasma

Biocompatibility, low toxicity, good mechanical properties, and high resistance to chemical corrosion render titanium and its alloys most suitable for implants [18,19]. N. Eliaz [18] provides a complex and comprehensive review of the corrosion of biomaterials, with historical background, evolution of materials, techniques for producing them, and the fundamentals of corrosion and tribology. Studies on the interaction between these materials and the human body have used simulated body fluid at pH 7.4 in electrochemical corrosion experiments [16,19,33,34,35]. In the present work, the electrochemical measurements have been performed in a typical 3-electrode cell, having the calomel electrode as a reference electrode and a Pt wire as an auxiliary electrode, whereas the respective stem samples were employed as a working electrode. The electrolyte was synthetic plasma that mimics the plasma of a healthy person (Table 3). The precursors were dissolved in distilled water by magnetic stirring for 8 h and filtered to remove any undissolved particles or impurities. The pH immediately after synthesis was 9.2 and it was set at 7.4 by adding 1.6 mL HCl 37%.

Instrumentation settings details: −1 V → 1 V potential range, 100 mV/s scanning rate for 5 cycles and a 1:8 sampling rate. The instrumentation used for the measurements was OrigaFlex potentiostat (OrigaLys, Rillieux-la-Pape, France). No smoothing was performed on the voltammograms. The peak intensity was determined by first manually drawing the baseline and then integrating each peak in OrigaMaster 5 software (OrigaLys, Rillieux-la-Pape, France).

The next step was to alter the synthetic plasma with glucose and urea in agreement with the biochemistry values at the presentation of the respective patient, shown in Table 4.

Since the present research considers three cases, diagnosed with AHT, cardiovascular disease (including dyslipidaemias), and chronic renal disease, we did not add cholesterol in the altered composition of the “healthy” synthetic plasma, but instead bore in mind that all their conditions generate inflammatory states [32,33,34,35,36,37,38].

In our simulation, the fragments of prostheses (dimensions: L = 20 mm, l = 11 mm and h = 6.56 mm) were immersed in 2 mL altered synthetic plasma and ultrasonicated for 7 h at 36 kHz, at a temperature of 37 °C. The electrochemical measurements were performed using the OrigaFlex potentiostat (OrigaLys, Rillieux-la-Pape, France) using DRP-150 microsensors from Dropsens (Metrohm DropSens, Vivero de Ciencias de la Salud, Spain). The DRP-150 microsensors are composed of the following: (1) a working electrode made of carbon; (2) a reference electrode made of silver; and (3) an auxiliary electrode made of platinum. Samples of 50 µL–100 µL were deposited on the microsensor and cyclic voltammetry (CV) was performed in the range [−1; 1.250] V with different scanning rates (50 mV/s, 100 mV/s, and 150 mV/s), for 5 cycles, and a 1:7 sampling rate. For the peak analysis, we chose the 3rd cycle, established the baseline, and determined the peak potential and peak intensity as current, using the dedicated software OrigaMaster 5 (Rillieux-la-Pape, France). Because of the small size of the samples, we switched from the typical 3-electrode assembly to a micro-electrode DRP-150 (Dropsens, Llanera, Spain). The OrigaLys potentiostat (Metrohm, France) was replaced by the Dropsens bipotentiostat, adapted for microsensor electrochemisty. Briefly, the micro-electrode (composed of a carbon working electrode, a Pt auxiliary electrode, and a Ag pseudo-reference electrode—all of them printed on a ceramic support) was inserted into a 3-channel slit connected to the bipotentiostat. A 50 µL sample was dropped and dispersed on the surface of the micro-electrode, fully covering it. The potential was swept between −1 V and 1 V with a 100 mV/s scan rate and a 1:7 sampling rate, for 5 cycles.

#### 2.3.2. Tissue in Synthetic Plasma

Five fragments (S1, …S4, soft tissue, and S5 bone with metal thin layer) remnant on the broken stem (Stem 1) were each immersed in 10 mL synthetic plasma and ultrasonicated for 15 min at 25 kHz to extract the components. Three pH-values were used: 7.3 (S1 and S2), 6.9 (S3 and S4), and 8.5 (S5).

#### 2.3.3. Surface Morphology, Surface Roughness, and Element Analysis

The surface morphology is a key factor for the osseointegration of implants, a mechanism occurring directly at the bone/metal alloy interface. The osteoconductive properties of surfaces enhance osseointegration with host tissues, i.e., the growth of bone on the surface of an implant [38,39,40,41,42]. Therefore, the characteristics of the surfaces of implants are highly important. Surface morphology and the element compositions of the stem samples were examined by energy dispersive X-ray spectroscopy (EDX), using a Hitachi TM3030 Plus tabletop scanning electron microscope (SEM), TM3030 Tabletop SEM (Hitachi, Tokyo, Japan/Hitachi Europe Ltd., Bucharest, Romania) equipped with a QUANTAX 70 EDS (Bruker, Billerica, MA, USA/Bruker Nano GmbH, Berlin, Germany) energy dispersive X-ray spectrometer.

**Figure 3:**
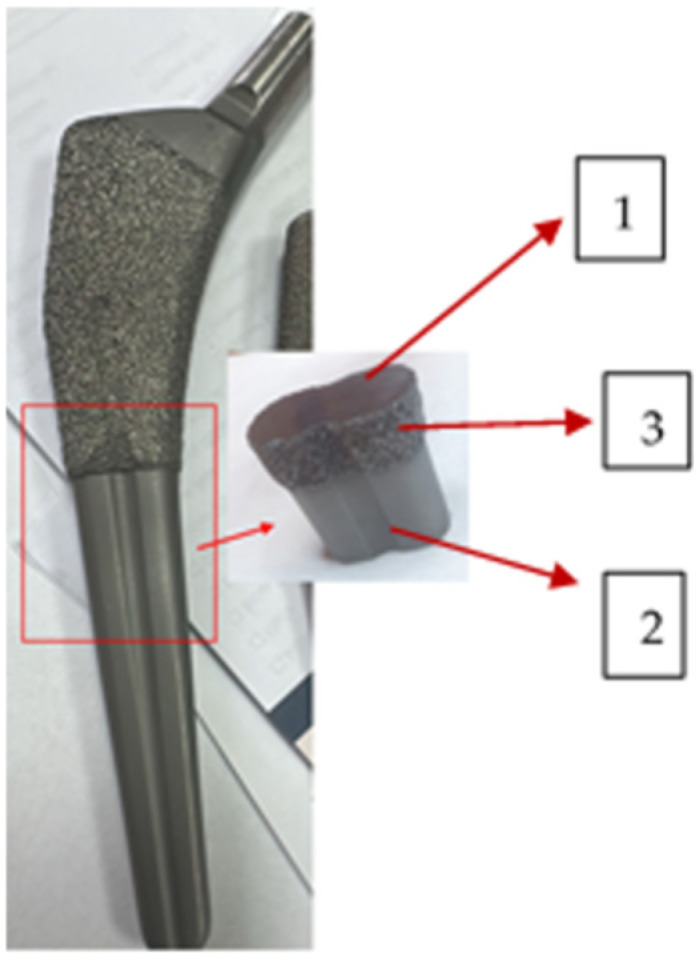
Cut of the stem: 1—cross section, substrate polished; 2—fine layer; 3—rough layer. The surface roughness was assessed by surface profilometry using a Veeco Dektak 150 profilometer (Bruker, Billerica, MA, USA/Bruker Nano GmbH, Berlin, Germany).

The SEM enables effective image analysis by combining two signals into a single image: the secondary electron (SE) signal that provides surface-rich information, and the backscattered electron signal that supplies compositional information. The linear calibration of the EDX system was carried out using the Cu-Kα (8.037 keV) and Cu-Lα (0.926 keV) lines recorded from a Cu standard sample. The EDX spectra of the samples were recorded over the 0–15 keV energy range with an accumulation time of 1200 s. In Figure 3, the image of a cut and polished zone is displayed, showing the analysed zones.

For each sample, the mean surface roughness (Ra) was calculated as the average of five mean roughness values obtained from line surface profiles recorded in different zones of the sample. Each line profile was acquired by scanning the surface with a 2.5 µm radius stylus pressed onto the sample surface with a force of 5.0 mg, moving at a speed of 0.1 mm/s over a distance of 10 mm. Height data were recorded with a scan step of 0.33 µm. A high-pass filter was applied to remove surface waviness from the line profiles.

#### 2.3.4. Histopathological Analyses of Soft Tissue Remnant on Stem 1

The slides were prepared following the usual flux: fixation in formalin 10%, trimming and transfer to labelled cassettes, histoprocessing with vacuum infiltration processor (Tissue-Tek Vip, Tokyo, Japan), and paraffin embedding (Sakura Tissue-Tek station, Tokyo, Japan), sectioning with a microtome (Leica RM 2245, Nussloch, Germany), transferring to microscope slides, and staining with haematoxylin + eosin (HE). Further on, the slides were examined with an Olympus BH-2 BHS microscope, (Tokyo, Japan) equipped with an Olympus SC50 camera (Munster, Germany) and a Cell Sense dedicated software (Entry 1.16, Build 15404, Olympus Corporation, Tokyo, Japan).

## 3. Results

### 3.1. Electrochemistry

#### 3.1.1. Stems in Synthetic Plasma

**Figure 4:**
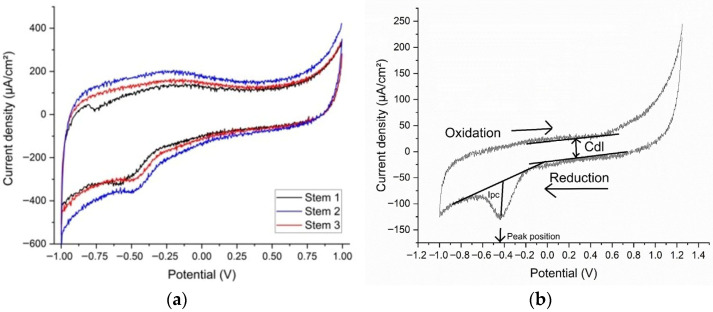
(**a**) The voltammograms and (**b**) the double-layer capacitance for cyclic voltammetry of samples.

The reactions corresponding to the reduction peak could be attributed to either HPO(OH)_2_ + 2H^+^ + 2e^−^ → H_2_PO(OH) + H_2_O (standard potential −0.5 V) present in the synthetic plasma, or S + 2e^−^ → S^2−^ (standard potential −0.48 V), which could be deposited as a residue on the prostheses. The voltammograms show that corrosion is not accelerated by the presence of glucose or urea. In the voltammetry, the −0.515 V potential is like the standard potential for the reduction in phosphide (H_3_PO_3_). Figure 4a,b show, respectively, the voltammograms and the double-layer capacitance, with the oxidation and reduction waves, the peak position (reduction potential), and the current density (Ipc) placed on the figure.

**Figure 5:**
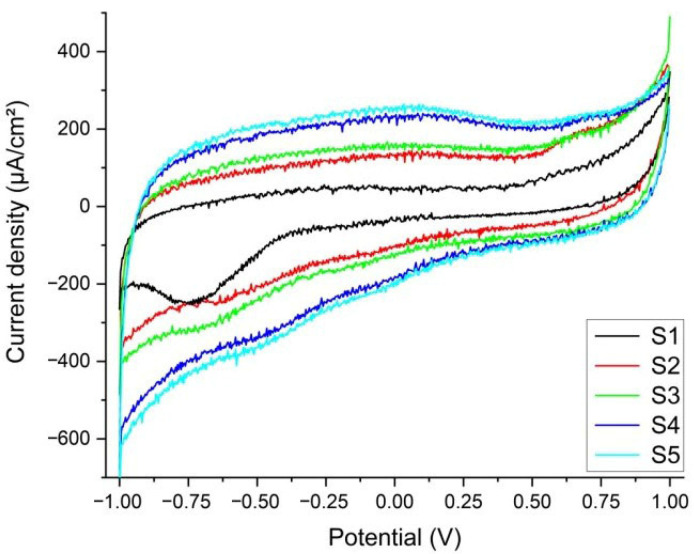
Voltammograms of the five tissue samples immersed in synthetic plasma. Only S1 has a visible reduction peak at −0.752 V. S5 (sample with a bone inclusion and a fine metal-like coating) demonstrates that a minute amount of vanadium has leached and is present in the extraction solution (standard potential at −0.255 V and the experimental potential of the reaction is −0.278 V).

The reactivity of the stems in altered synthetic plasma is given mainly by the presence of glucose and urea. Several studies refer to glucose in inflammatory synthetic environment [14,16,35,43,44,45], urea being mentioned rarely [14,45]. In our study, based on three real cases, the double-layer capacitance (Cdl) shows that the combination of glucose and urea, mimicking real-life underlying conditions of the patient, have produced a decrease in the adsorption/desorption process that led to a reduction reaction evident in the CV at −0.500 V. Aside from reference [44], a study on Gold 21k in artificial sweat (containing glucose and urea), the materials involved are Ti-based alloys. In our experiments, even though the intensities of the peaks are low, the occurrence of the reduction peaks shows that the presence of glucose and urea have a direct influence on the Ti-based prosthesis (Figure 5). Previous publications [13,14,28] suggest that changes in the biological environment would lead to changes in the surfaces of the implants immediately after implantation.

#### 3.1.2. Tissue in Synthetic Plasma

From the CV of the five tissue samples (S1…S5), in the first cycle, at a 100 mV/s scanning rate, only S1 has a visible reduction peak at −0.752 V (Figure 5), corresponding to the reaction FeCO_3_(s) + 2e^−^ → Fe(s) + CO_3_^2−^ (standard potential at −0.756 V) [46]. Ferrous carbonate is a porous corrosion product that forms on the surface of the prosthesis through the reaction of the CO_3_^2−^ ions dissolved in the plasma with the Fe in the alloy (see EDX and Figure 4a,b). It acts as protection against further corrosion by installing a diffusion barrier and slowing down the leaching of the metal [17,18]. In comparison with S1, the other samples do not seem like there is leaching of the metals at this scanning rate. The reduction in the scanning rate down to 10 mV/s shows that, for S1, the iron carbonate is consumed in the initial cycles, missing in the subsequent, low scanning rate, cycles. For the other samples, only S5 (sample with a bone inclusion and a fine metal like coating) demonstrates that a minute amount of vanadium has leached and is present in the extraction solution (V^3+^ + e^−^ → V^2+^) with a standard potential at −0.255 V, and the experimental potential of the reaction is −0.278 V for S5 [9,17]. For the rest of the samples, the potential corresponds to the reduction in the dissolved oxygen to O_2_ + e^−^ → O_2_^−^ (standard potential is at −0.33 V whereas our experimental potential is determined to be −0.334 V).

Diabetic patients with Ti6Al4V prostheses, especially TH and femoral ones, can expect earlier loosening and failure than the non-diabetic ones because of an increased coefficient of friction [35]. This seems to be true for Case 2-M, who fractured his femur and stem, and is expected for F1 and F2.

### 3.2. Surface Morphology, Surface Roughness, and Element Analysis

**Figure 6:**
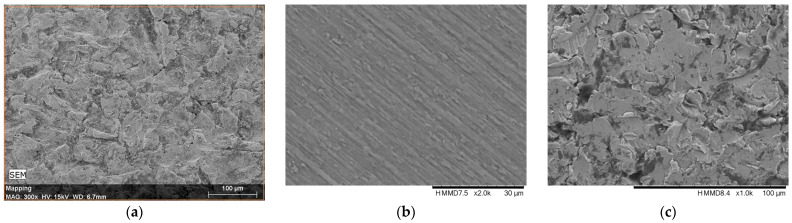

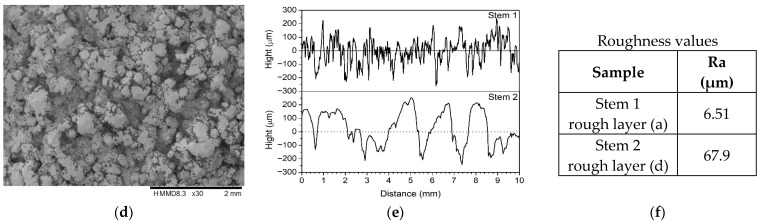
SEM images of surfaces corresponding to the following: (**a**) the broken, retrieved Stem 1 (Figure 2, broken end), (**b**) the polished substrate of Stem 2 (Figure 3, label 1), (**c**) the fine layer of Stem 2 (Figure 3, label 2), and (**d**) rough layer of Stem 2 (Figure 3, label 3); (**e**,**f**) profilometry measurements and roughness values on coatings of Stem 1 and Stem 2, respectively, corresponding to SEM images (**a**,**d**) in this figure.

Figure 6a–d show the SEM images of surfaces corresponding to the following: (a) the broken, retrieved, Stem 1 (Figure 2, broken end), (b) the polished substrate of Stem 2 (Figure 3, label 1), (c) the fine layer of Stem 2 (Figure 3, label 2), and (d) the rough layer of Stem 2 (Figure 3, label 3). The profilometry results are presented in Figure 6e,f. From Figure 6, it is observed that the three layers of the Stem 2 have different structures, the roughest being the external one (Figure 3, label 3, and Figure 6d). The surface of Stem 1 corresponds to the rough layer of Stem 2 (coating). However, the morphology of the coating of Stem 1 is most like that of the fine layer (Figure 6c and Figure 3, label 2). The diabetic and uremic environment of Stem 1 during 7 y 6 m, accompanied by motion, led to wear by corrosion and friction. At the interface between the cancellous bone and the surface of the implant, cells in the healing tissue can also produce corrosion [14]. Also, we bear in mind that Stem 1 and Stem 2 are different versions from the same producer, and we have no information of the initial image of the surface of Stem 1. However, the plasma spray technique to deposit HAp on Zimmer Biomet stems has been in use for several decades.

EDX spectra show that the stems’ substrates are basically built of Ti-6Al-4V including small amounts of iron and carbon, with coating layers of HAp (Table 5 and Table 6), thus supporting the CV results (Figure 4).

The EDX analysis is particularly useful to understand the probability of metal ion migration in the body. It is practically impossible to neatly separate the layers between each other, and from the substrates, because deposition techniques aim for adhesion, and hence for interdiffusion at the interfaces [9,34,35,39]. An important remark is the small amount of phosphorus, especially in the layer of Stem 1. Calcium and phosphorous are rapidly absorbed in the healing process [14,38,41,47]. Also, there is a great difference between the vanadium concentration in the substrate of Stem 1 and its layer. We consider vanadium leached after the stem has broken in M’s femur, and view the electrochemistry measurements and supporting publications [5,17,33,35,45].

### 3.3. Histopathology Analyses of Soft Tissue Remnant on Stem 1

**Figure 7:**
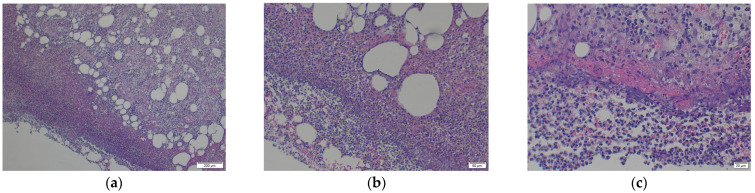
The histopathology analysis (**a**–**c**) shows a pronounced effect of lipids in the sample extracted from Stem 1, in agreement with the biochemistry blood tests of patient M: (**a**) Loss of histological details of subcutaneous tissue (necrosis). Numerous neutrophils intermixed with macrophages and a few lymphocytes are present, supporting the numerous small new capillaries (granulation tissue); (**b**) Additional numerous extravasated erythrocytes and several fragments of inflammatory cells (cellular dust) are very evident in the sections; (**c**) Subcutaneous tissue detail: 80% intact, and degenerated neutrophils, intermixed with red blood cells (purulent inflammation and discrete haemorrhage are present); Red fibrillar to homogenised proteinaceous material is evident, closely related to inflammatory reaction described earlier; Lymphocytes and macrophages with small capillaries, are completing the entire view of the lesion.

The histopathology analysis (Figure 7a–c) shows a pronounced effect of lipids in the sample extracted from Stem 1, in agreement with the biochemistry blood tests of the patient. Severe subcutaneous reaction characterised by cellulitis, panniculitis, and myositis is present. There is no evidence of a neoplastic aspect or other pathogens microscopically visible. However, the loss of histological details of subcutaneous tissue indicates necrosis. Numerous neutrophils intermixed by macrophages, and a few lymphocytes, are present, supporting the abundance of small new capillaries (granulation tissue). The density of inflammatory cells, including neutrophils, macrophages, and fibroblasts, is approximately 80 cells per high-power field under high magnification. Direct corrosion by macrophages, monocytes, and osteoclasts has been reported in culture media [35].

## 4. Discussion

Understanding bones, their quality, damage, and ways of recovering, including prosthetics of all sorts, has demanded a great effort for a long time. Orthopaedics surgery involves various kinds of implants, whose interfaces with the bone tissues, and their surroundings, are dramatically influenced by the characteristics of the implant, e.g., geometry, material, surface morphology, topology, mechanical, physical, and chemical properties [1,2,3,4,5,6,7,8,9], and, by interaction with the living body, present dysfunctions of important internal organs, such as pancreas, kidneys, parathyroid, liver, and heart [10,11,12,13,14,15]. Three cases were reported, two women, F1 and F2, and one man, M, of different ages, undergoing HA (F1-primary, F2, and M-revision) with uncemented Ti-alloy (Ti6Al4V) and sharing cardiovascular disease, diabetes, and kidney disease. F2 and M also shared dyslipidaemia. Two stems (one broken in M’s femur and one unused), and remnant tissue on the retrieved stem, were investigated by electrochemistry, showing that the reactivity of the stems in altered synthetic plasma is given mainly by the presence of glucose and urea. One sample with a bone inclusion coated with a fine metal-like film demonstrated that a minute amount of vanadium (V) had leached and was present in the extraction solution at the experimental potential of the reaction of −0.278 V. A leak of V is supported by EDX analysis, showing a remarkable difference between the V concentration (at. %) in the broken stem and the unused one. Thus, electrochemistry and surface analysis indicate the migration of vanadium from the alloy onto bone tissue in the fractured case within six months, consistent with the elevated vanadium reported in the serum in other studies. The high variation coefficients for the measurements of the biological samples (tissue in synthetic plasma) can be explained by the complex structures of the biological materials, the different material pre-treatment (synthetic plasma vs. natural blood plasma), different electrode preparation (screen printed electrodes vs. three-electrode standard assembly), and the presence of corrosion products as impurities. Five repeated cyclic voltammetries were recorded for each measurement, which indicates a variation coefficient of 5.88% for the stems. For the measurements of the tissue in synthetic plasma, we calculated a variation coefficient of 21.21%. The diabetic and uremic environment of M’s broken stem, during 7 y 6 m, accompanied by motion, led to wear by corrosion and friction. At the interface between the cancellous bone and the surface of the implant, cells in the healing tissue can also produce corrosion. Chemical corrosion driven by abnormal body chemistry weakens the surface, promoting crack initiation, thus interacting with mechanical wear. These processes are difficult to separate in vivo. The histopathology analysis proves a density of inflammatory cells of, approximately, 80 cells per high-power field under high magnification. Those include neutrophils, macrophages, and fibroblasts that contribute to corrosion. Direct corrosion by macrophages, monocytes, and osteoclasts has been reported in culture media. In general, inflammation around implants may reflect biological reaction or bacterial or aseptic causes, leading to complications of case management. The SEM-EDX analysis of the outer layers of the stems demonstrates that hydroxyapatite loses its compositions in time, owing to the high absorption of phosphorus and calcium during healing.

The present case underscores the broader principle that patient-specific factors significantly shape THA outcomes beyond the surgical technique alone. Recent meta-regression analyses by our group demonstrated that variables such as age, metabolic status, incision length, intraoperative blood loss, and time to mobilisation are robust predictors of postoperative functional recovery and complication risk across both minimally invasive and conventional approaches [48,49]. These findings support the hypothesis that systemic factors—including comorbidities such as diabetes, dyslipidemia, or impaired bone biology—may modify peri-implant tissue response and contribute to mechanical or biological complications, as observed in the current case.

Patients with dyslipidaemias, cardiovascular disease, and chronic kidney disease are at higher risk of fractures, especially hip fractures, as compared to healthy ones, because of changes in bone turnover, mineralisation, and volume. Although Ti-alloys are biocompatible, comorbidities degrade bone–implant interfaces, suggesting reduced implant lifespan in affected patients.

The three cases we reported here are complex examples of diabetes, cardiovascular disease, kidney disease, and dyslipidaemias, all requiring HA (either primary, or revision) with Ti6Al4V femoral stems. The enormous number of reviews on the material, the processing of prostheses, the interplay between those, and patients’ underlying conditions, give important insights into the necessity of a more holistic diagnostic for orthopaedic patients. P. Anagnostis et al. [13] touch upon a hot live, although underrepresented, subject, i.e., dyslipidaemias and osteoporosis. The review by M. Prestat and D. Thierry [35], claimed in 2021 as the first review “focused specifically on the topic of inflammation”, examines the research conducted over forty years on the in vitro studies on titanium corrosion under simulated inflammation conditions, involving materials science, corrosion, and prosthetics. In the same trend, we have already mentioned the work by N. Eliaz [18]. This only strengthens the high importance of these studies, and reviews, for advanced knowledge in the long term. To summarise, the bone–Ti-alloy interaction follows two strands: (1) the bone health and (2) the alloy characteristics, and the interface surfaces. Body fluids are permanent carriers of various chemical, and biochemical, compounds, released either through the metabolic functions of the patient or from the interaction between those and the materials of prostheses, also accounting for the mechanical influence. Systemic comorbidities, such as diabetes, dyslipidaemia, and renal dysfunction, though controlled, can degrade, in the long term, the bone–implant interface and bone quality, increasing the risk of loosening, fracture, or earlier prosthesis revision, even for biocompatible materials like Ti-alloys. Also, it must be taken into account that aged patients with long-lived prostheses (e.g., F2) could have been free of comorbidities at their primary HA and develop systemic comorbidities much later, with ageing. There are limitations of this present work, and we do not claim its universality, because it is a three-case report and not a cohort study. Also, the ex vivo accelerated testing and plasma model have simplified the long-term, complex in vivo conditions. Larger studies have been published in refs. [10,11,12,13,14,15,35], to cite a few, on which we called in support of this present work. Those studies have been the basis of building the models for our ex vivo experiments. Other limitations of this experimental work consist in considering only femoral stems, Ti6Al4V alloy only, from Zimmer Biomet solely. A brief review supports the findings, but the work remains essentially a case report and not a review. Further studies are recommended to quantify risks and guide implant selection and management in practice.

Suggestions for future clinical work would be to optimise metabolic control (previous to and following HA), at least to monitor chemical corrosion risk and improve bone health, understand the causes of the prosthesis failure and act in a personalised implant strategy, monitor high-risk patients more closely, and eventually prescribe measures of prevention with respect to bone quality preservation.

## 5. Conclusions

In this three-case report, we observed that patients with diabetes, dyslipidaemia, cardiovascular disease, and chronic kidney dysfunction demonstrated alterations at the bone–implant interface despite the use of biocompatible Ti6Al4V stems. Electrochemical and surface analyses indicated early vanadium migration and corrosion phenomena under metabolically altered conditions. These findings suggest that systemic comorbidities may accelerate biological and chemical degradation processes around Ti-alloy implants. Further clinical and experimental work is needed to quantify these risks and to improve implant selection and postoperative management in metabolically compromised patients.

## Figures and Tables

**Table 1:** Summary of the characteristic of the three patients.

Case No.	Sex	Age [Years]	HA/Cause		Type of Prosthesis	Underlying Conditions	Remarks
			Primary	Revision Time [Years]			
1-F1	F	35+	Yes; basicervical femoral neck fracture; unspecified fall; hemiarthroplasty; periprosthetic fracture	-	Bipolar, Zimmer Biomet, Ti6Al4V, stem and cable fixation	Type 1 diabetes; renal failure end stage; essential hypertension	Insulin dependency, dialysis dependency
2-F2	F	80+		Yes; 15; periprosthetic fracture; unspecified fall	Taper lock complete, Zimmer Biomet, Ti6Al4V stem	High blood pressure (AHT); dyslipidaemia; hyperglycaemia; azotaemia; uraemia hypothyroidism	-
3-M	M	65+		Yes; 7; periprosthetic fracture; unspecified fall	Taper lock complete, Zimmer Biomet, Ti6Al4V stem	AHT third degree, congestive heart failure, old myocardial infarction, coronary artery disease (CAD), atrioventricular block (AV) first degree, right bundle branch block (RBBB), dyslipidaemia (mixed hyperlipidaemia), azotaemia, uraemia	Fractured stem left in place 6 months till revision; bone fragment with thin metal layer; periprosthetic tissue with cellulitis, panniculitis, myositis

**Table 2:** Characteristics of samples.

Sample Name	Type	Condition	Source	Size
Stem 1—fragment from the broken end	Solid, Ti-alloy + HAp, femoral stem	Broken approx. perpendicular to its longitudinal axis, 7 years used unbroken, 6 months used broken	Femur of patient M, aged over 65 years with cardiovascular disease, type 2 diabetes, dyslipidaemia, chronic renal disease, azotaemia, uraemia	L = 20 mm, l = 11 mm and h = 6.56 mm
Stem 2—fragment from the distal end	Solid, Ti-alloy + HAp, femoral stem	Unutilised	Unutilised	L = 20 mm, l = 11 mm and h = 6.56 mm
Stem 3—fragment from the distal end	Solid, Ti-alloy + HAp, femoral stem	Unutilised	Unutilised	L = 20 mm, l = 11 mm and h = 6.56 mm
Synthetic plasma (Table 3)	Liquid	Synthesised		50 µL/sample
3 × soft tissue fragments	Solid	Ex vivo direct	Surface of Stem 1	1.5 cm^2^ 1.098 g each
Bone tissue fragment with thin metal layer	Solid	Ex vivo direct	Surface of Stem 1	1.75 cm^2^ 2.532 g

**Table 3:** Composition of the synthetic plasma corresponding to a healthy person.

Compound	NaCl	CaCl_2_	KCl	MgSO_4_	NaHCO_3_	Na_2_HPO_4_	NaH_2_PO_4_
Mass [g]	6.80267	0.20370	0.40099	0.10153	2.20212	0.12780	0.02644

**Table 4:** Alteration of “healthy” synthetic plasma with glucose and urea in agreement with the biochemistry values at the presentation of the respective patient.

Compound Added to the “Healthy” Synthetic Plasma	Concentration in “Healthy Synthetic Plasma” [mg/dL]	Patient
Glucose	266	F1
Urea	119	
Glucose	118	F2
Urea	85	
Glucose	95	M
Urea	50	

**Table 5:** Element analysis of Stem 1, substrate, and external layer.

Stem 1, Layer		Stem 1, Substrate	
Element	At. %	Element	At. %
Titanium	24.05 ± 1.73	Titanium	75.09 ± 2.91
Aluminium	10.53 ± 0.70	Aluminium	7.02 ± 0.27
Vanadium	0.82 ± 0.09	Vanadium	2.79 ± 0.14
Oxygen	55.22 ± 4.95	Carbon	8.50 ± 0.41
Calcium	0.81 ± 0.07	Oxygen	5.60 ± 0.42
Phosphorus	0.001 ± 0.00	Nitrogen	0.99 ± 0.10
Carbon	5.73 ± 0.48		
Silicon	1.00 ± 0.09		
Sodium	0.99 ± 0.10		
Potassium	0.31 ± 0.04		
Iron	0.30 ± 0.05		
Magnesium	0.18 ± 0.04		
Molybdenum	0.05 ± 0.03		

**Table 6:** Element analysis of Stem 2.

Stem 2, Substrate		Stem 2, Fine Layer		Stem 2, Rough Layer	
Element	At. %	Element	At. %	Element	At. %
Titanium	72.53	Titanium	60.29	Titanium	37.18
Aluminium	7.86	Aluminium	3.54	Aluminium	3.41
Vanadium	2.35	Vanadium	1.90	Vanadium	1.30
Silicon	0.13	Silicon	3.73	Silicon	0.35
Iron	0.07	Iron	0.76	Iron	0.08
Oxygen	7.41	Calcium	0.61	Calcium	0.10
Nitrogen	0.91	Phosphorus	0.001	Phosphorus	0.04
Carbon	8.74	Oxygen	24.92	Oxygen	28.49
		Carbon	3.59	Carbon	28.18
		Sodium	0.51	Sodium	0.67
		Magnesium	0.12	Magnesium	0.09
				Chlorine	0.07
				Potassium	0.04

## Data Availability

The original contributions presented in this study are included in the article. Further enquiries can be directed to the corresponding authors.
